# Implementation of a group-based diabetes prevention program within a healthcare delivery system

**DOI:** 10.1186/s12913-019-4569-0

**Published:** 2019-10-15

**Authors:** Kristen M. J. Azar, Catherine Nasrallah, Nina K. Szwerinski, John J. Petersen, Meghan C. Halley, Deborah Greenwood, Robert J. Romanelli

**Affiliations:** Palo Alto Medical Foundation Research Institute, 795 El Camino Real, Ames Building, Palo Alto, CA 94301 USA

**Keywords:** Diabetes prevention program, Diabetes mellitus; program evaluation, Healthcare system, Health promotion, Qualitative evaluation

## Abstract

**Background:**

Group-based Diabetes Prevention Programs (DPP), aligned with recommendations from the Centers for Disease Control and Prevention, promote clinically significant weight loss and reduce cardio-metabolic risks. Studies have examined implementation of the DPP in community settings, but less is known about its integration in healthcare systems. In 2010, a group-based DPP known as the Group Lifestyle Balance (GLB) was implemented within a large healthcare delivery system in Northern California, across three geographically distinct regional administration divisions of the organization within 12 state counties, with varying underlying socio-demographics. The regional divisions implemented the program independently, allowing for natural variation in its real-world integration. We leveraged this natural experiment to qualitatively assess the implementation of a DPP in this healthcare system and, especially, its fidelity to the original GLB curriculum and potential heterogeneity in implementation across clinics and regional divisions.

**Methods:**

Using purposive sampling, we conducted semi-structured interviews with DPP lifestyle coaches. Data were analyzed using mixed-method techniques, guided by an implementation outcomes framework consisting of eight constructs: acceptability, adoption, appropriateness, cost, feasibility, fidelity, penetration, and sustainability.

**Results:**

We conducted 33 interviews at 20 clinics across the three regional administrative divisions. Consistencies in implementation of the program were found across regions in terms of satisfaction with the evidence base (acceptability), referral methods (adoption), eligibility criteria (fidelity), and strategies to increase retention and effectiveness (sustainability). Heterogeneity in implementation across regions were found in all categories, including: the number and frequency of sessions (fidelity); program branding (adoption); lifestyle coach training (adoption), and patient-facing cost (cost). Lifestyle coaches expressed differing attitudes about curriculum content (acceptability) and suitability of educational level (appropriateness). While difficulties with recruitment were common across regions (feasibility), strategies used to address these challenges differed (sustainability).

**Conclusions:**

Variation exists in the implementation of the DPP within a large multi-site healthcare system, revealing a dynamic and important tension between retaining fidelity to the original program and tailoring the program to meet the local needs. Moreover, certain challenges across sites may represent opportunities for considering alternative implementation to anticipate these barriers. Further research is needed to explore how differences in implementation domains impact program effectiveness.

## Contributions to the literature

- Little is known about the integration of the Group-based Diabetes Prevention Programs (DPP), aligned with recommendations from the Centers for Disease Control and Prevention, in healthcare systems. We assessed the implementation and local adaptations in structure and design of a DPP within a large healthcare delivery system in Northern California, across three geographically distinct regional administration divisions of the organization within 12 state counties, with varying underlying socio-demographics.
- We found instances of both consistency and variation in implementation of the program across three geographic regions, with very different underlying sociodemographic characteristics.
- The findings of our study expose a dynamic and important tension between the attempt to retain fidelity to the original evidence-based program and the need to tailor the program to meet the local needs of the organization, distinct patient populations, and the clinical context.

## Background

According to the Centers for Disease Control and Prevention (CDC), around 70% of Americans are overweight or obese, of which 9.5% have diagnosed type 2 diabetes (T2D) [1]. Further, an estimated 84 million people in the United States (U.S.) have prediabetes, a condition that increases the risk of T2D and cardiovascular diseases (CVD) and in some instances may be reversible [2]. Given the increased direct and indirect costs of T2D [3], and its adverse outcomes on the individual’s quality of life [4], opportunities for prevention are of paramount importance for the long-term health of the American population and the efficiency of the healthcare system [1].

Research has shown that lifestyle interventions promoting moderate physical activity and healthy eating habits positively impact long-term weight management and prevention of T2D [2, 5]. The Diabetes Prevention Program (DPP) was a landmark randomized controlled trial (RCT) that established the gold standard for T2D prevention in the U.S [6]. The trial demonstrated that a predominantly one-on-one lifestyle behavioral intervention, promoting weight loss through healthy eating habits and increased physical activity, reduced CVD risk factors [7–9] and lowered the risk of developing T2D by 58% relative to placebo, [6, 10–12] irrespective of age, gender, race, and ethnicity [7, 13, 14]. Given the efficacy of the DPP intervention [8, 15–17] in 2010 Congress authorized the CDC to establish the National DPP to facilitate large-scale dissemination and implementation of effective lifestyle interventions modeled after the original DPP intervention [18]. As a result, organizations across the nation began to offer group-based versions of the DPP, in community and clinical settings [15, 17, 19]. Numerous subsequent studies [20–22] of group-based DPP translations in primarily community-based settings, have demonstrated the effectiveness of these adapted programs in reducing the T2D risk among participants.

The CDC established the National Diabetes Prevention Recognition Program (DPRP) to ensure quality and effectiveness of evidence-based DPP translations. CDC guidelines leave much room for variation in the format of program delivery. While the National DPRP provides standards and basic criteria that must be met by all CDC-recognized programs, there is still room for variation allowing for organizations to adapt aspects of the program to a particular setting. There is little information on the implementation of this program and the ways in which it is adapted to meet local needs. Research is needed to understand the extent of this variation in the context of real-world implementation and to explore the balance between the importance of fidelity to the program and the need to tailor the program to specific populations and delivery environments in order to optimize outcomes.

In 2010, a group-based DPP known as the Group Lifestyle Balance™ (GLB) program was implemented within a large healthcare delivery system in Northern California, across three geographically distinct regional administration divisions of the organization within 12 state counties, with varying underlying sociodemographics. The regional divisions implemented the program independently, allowing for natural variation in its real-world integration. The aim of this study was to assess the implementation of a DPP in this healthcare system and, especially, its fidelity to the original GLB curriculum and potential heterogeneity in implementation across clinics and regional divisions. This natural experiment provides a unique opportunity to explore variation in the integration and adaptations of a group-based DPP (hereafter group-DPP). Findings may inform national diabetes prevention guidelines for ensuring both program fidelity while allowing for appropriate local adaptations.

## Methods

This was a qualitative, descriptive study. We conducted semi-structured interviews with DPP lifestyle coaches (LCs). LCs are existing clinical staff who facilitated the face to face program sessions to program participants. They are typically dieticians or nurses who receive additional training in order to facilitate the DPP program. Data were analyzed using mixed-method techniques, guided by an implementation outcomes framework consisting of eight constructs as described below.

### Setting

This study was conducted at Sutter Health, a large multi-specialty healthcare delivery system in northern California. Sutter Health serves approximately 3 million patients per year in more than 100 communities across 5 regional administrative divisions. Sutter Health is a mixed-payer, fee-for-service (FFS) provider organization, which contracts with various commercial payers, as well as the Centers for Medicare and Medicaid Services. Sutter Health’s group-DPP, the GLB program (15, 16), was implemented at a total of 20 clinic sites between 2010 and 2016. The GLB was developed by the University of Pittsburgh [7, 8, 10, 15, 23]. The curriculum is composed of 16 core sessions (12 weekly intensive core sessions and 4 biweekly transition sessions), followed by 6 monthly maintenance sessions (i.e. post-core) [10, 15, 24]. The program emphasizes on goal setting and planning, problem solving and social support, enhancing motivation to engage in healthy lifestyle practices, as well as self-monitoring of physical and calorie intake using a weekly food and physical activity tracker [10, 15]. During the maintenance sessions, skills learned during the core sessions are reinforced, and cognitive and behavioral strategies are introduced for long term-weight management [15].

### Conceptual framework

**Fig. 1:**
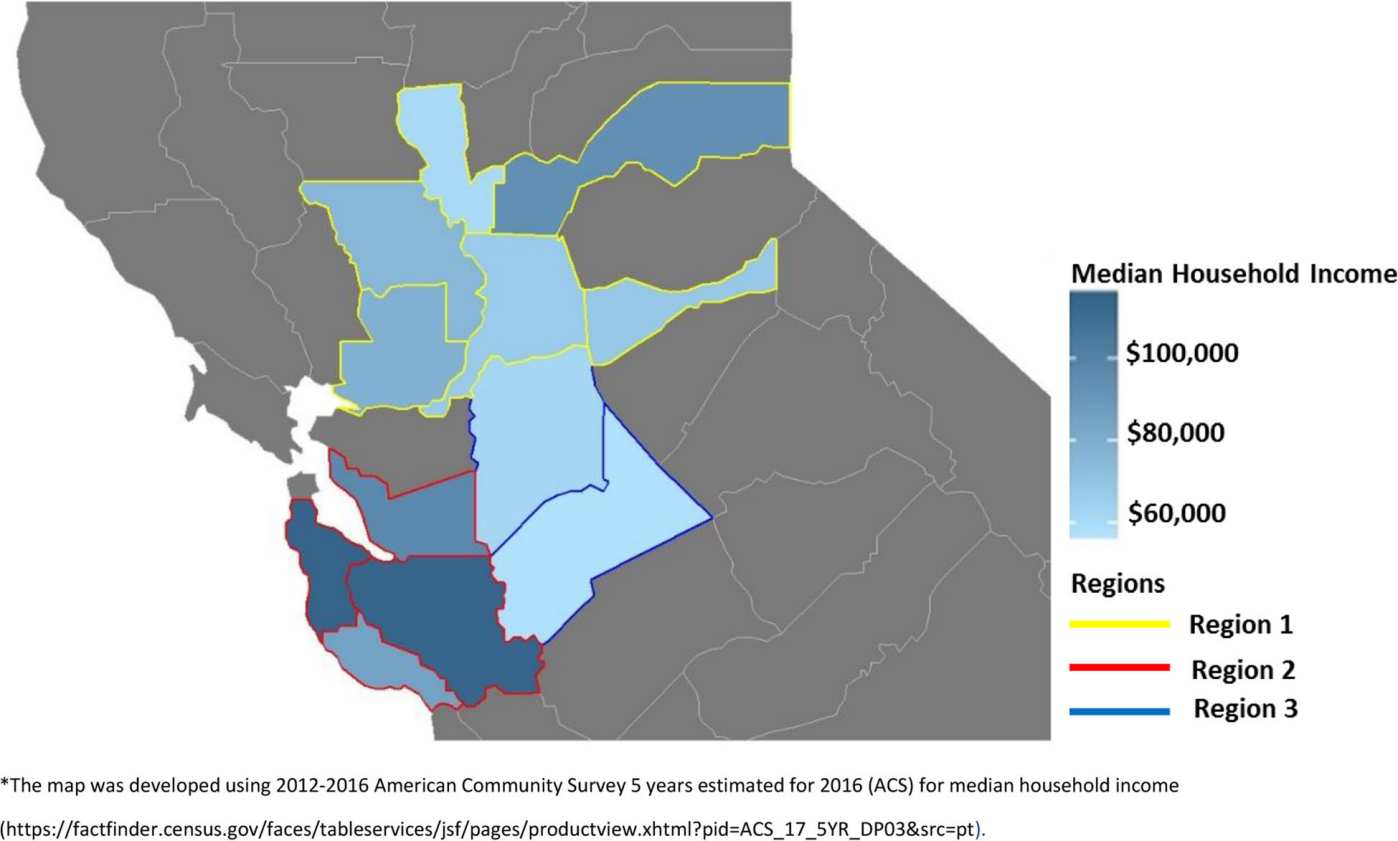
Median Household Income for California Counties with GLB clinics (2016)

We examined the implementation of the program across three geographical distinct regional administrative divisions within a single healthcare system (Fig. 1). We examined the consistency and variability in implementation across clinic sites using the implementation outcomes framework developed by Proctor et al. 2011 [25]. This framework puts forth the concept of “implementation outcomes” as distinct from service system and clinical treatment outcomes and proposes a heuristic consisting of eight conceptually distinct but interrelated implementation outcomes: adoption, penetration, acceptability, appropriateness, feasibility, fidelity, implementation cost and sustainability [25] (Table 1).

**Table 1:** Proctor et al. (2011) Implementation Domains

Adoption	The intention, initial decision, or action to try or employ an innovation or evidence-based practice
Penetration	The integration of a practice within a service setting and its subsystems
Acceptability	The perception among implementation stakeholders that a given treatment, service, practice, or innovation is agreeable, palatable, or satisfactory
Appropriateness	The perceived fit, relevance, or compatibility of the innovation or evidence-based practice for a given practice setting, provider, or consumer; and/or perceived fit of the innovation to address a particular issue or problem
Feasibility	The extent to which a new treatment, or an innovation, can be successfully used or carried out within a given agency or setting
Fidelity	The degree to which an intervention was implemented as it was prescribed in the original protocol or as it was intended by the program developers
Costs	The cost impact of an implementation effort
Sustainability	The extent to which a newly implemented treatment is maintained or institutionalized within a service setting’s ongoing, stable operations

### Interview protocol and procedures

We used purposive sampling to identify program LCs within each region and clinic site. Study team members (CN and NKS) first contacted program managers at each region by phone to explain the aim of the study and gather data on the features of the program, number of clinics offering the program, and names and contact information of LCs at each clinic. Then, current and previous program LCs were contacted by email to explain the aim of the study and to schedule semi-structured in-person interviews with them. Interviews were conducted at 20 clinic sites across the three regions between November 2017 and March 2018 by two research associates trained in qualitative research (CN and NKS).

Interviews lasted between 30 and 90 min. Interviewers used a semi-structured interview guide including both close-ended and open-ended questions covering the following: 1) LCs’ demographic characteristics; 2) characteristics of program clinic site; 3) eligibility and recruitment of program participants; 4) implementation and characteristics of the program; 5) maintenance and changes made over time; and 6) outcomes of the program. The interview guide was pilot tested and edited accordingly with a LC who previously provided the program at one of the clinic sites. Interviews were audio recorded using encrypted voice recorders and were then transcribed verbatim. In addition, study team members took thorough notes of all the information provided by participants and collected program flyers from each clinic sites. All study activities were reviewed and approved by the healthcare organization’s Institutional Review Board.

### Analytical approach

We utilized multiple methods to ensure the validity and reliability of the results reported below. First, after completing the interviews with LCs, the study team conducted a document review of all the notes taken during the interview as well as information included in the program flyers collected from clinic sites and information provided by site managers to confirm some of the accuracy of the information provided by LCs. Next, responses to close-ended questions included in the interview guide were analyzed quantitatively on STATA 16 using descriptive statistics (see Table 2). Responses to open-ended questions were analyzed thematically using a combination of deductive and inductive approaches in Dedoose, a multifunctional mixed-methods platform [26]. To identify inductive themes, three members of the study team (JJP, CN, NKS) reviewed 1233 transcribed pages of interviews and independently created a preliminary set of emergent codes characterizing the key themes discussed in the interviews. These codes were then reviewed together by the study team and revised, and organized into a structured codebook including a total of 68 codes. Three coders (CN, NKS & JJP) independently applied a total of 15,698 codes in Dedoose.

**Table 2:** Lifestyle Coaches Characteristics

Demographic Characteristics	Region 1 *N* = 10	Region 2 *N* = 21	Region 3 *N* = 2	Overall *N* = 33
Age				
Mean (SD)	43.5 (11.7)	49 (7.4)	54 (17)	47.5 (9.6)
(Min; Max)	(27; 60)	(34; 61)	(42; 66)	(27; 66)
Gender				
Female, n (%)	10 (100%)	20 (95.2%)	2 (100%)	32 (97%)
Current Occupation/Title				
Registered Dietitian	9 (90%)	20 (95.2%)	2 (100%)	31 (93.9%)
Registered Nurse	1 (10%)	1 (4.8%)	0 (0.0%)	2 (6.1%)
Certifications				
Certified Diabetes Educator	2 (20%)	16 (76%)	0 (0.0%)	18 (54.5%)
Certified Health Educator	8 (80%)	0 (0.0%)	1 (50%)	9 (27.3%)
Years Working at Sutter Health				
Mean (SD)	4.7 (2.5)	9.8 (6.5)	14.3 (8.1)	8.5 (6.1)
Range (Min; Max)	(1; 8)	(2; 29)	(8.5; 20)	(1; 29)
Years Facilitating GLB				
Mean (SD)	2.9 (2.2)	3.2 (1.8)	4 (4.2)	3.1 (2)
Range (Min; Max)	(0.25; 7)	(1; 6)	(1; 7)	(0.25; 7)
Type of Training Received				
Formal -University of Pittsburgh	1 (10%)	13 (61.9%)	2 (100%)	18 (51.4%)
Formal – Not University of Pittsburgh	0 (0.0%)	1 (4.8%)	0 (0.0%)	1 (2.9%)
Online Training	0 (0.0%)	4 (19.1%)	0 (0.0%)	4 (11.4%)
Peer-to-Peer	8 (80%)	3 (14.3%)	0 (0.0%)	11 (31.4%)
Don’t Know	1 (10%)	0 (0.0%)	0 (0.0%)	1 (2.9%)

When coding was complete, the team reviewed the data by code to identify the relevant implementation outcomes based on our conceptual framework. Inductive codes were assigned to relevant outcomes based on their definitions (see Table 1) using a team-based consensus approach. We aligned our inductively-derived thematic codes with the relevant constructs from the conceptual framework to structure our findings. For example, excerpts coded as “content/characteristics” and “background evidence of program” are reported as elements of acceptability, “program visibility” and “experience/training” elements of adoption, and “strategies for change” of sustainability. We then identified exemplary quotes to illustrate the key ideas grouped by the eight implementation outcomes.

## Results

The group-DPP is offered within three of five regional administrative division of the healthcare system (Fig. 1). Regions differed in underlying sociodemographic characteristics (Fig. 1) and the sociodemographic characteristics of program participants (Additional file 1). Region 1, the largest geographically of the three, spans seven counties, with an underlying racial/ethnic minority population of 23% Hispanic, 13% Asian, and 8% Black/African American [27], and a median household income of $73,439 [28]. Region 2 covers five counties, has a higher average household income of $106,489 [28] and different demographic with a larger Asian population (29%) [27]. Region 3’s catchment area includes three counties, which includes a larger Hispanic population (42% Hispanic) [27] compared to the other two regions and the lowest average household income of $60,170 [28].

All LCs agreed to participate in the study. A total of 33 LCs were interviewed, representing 20 clinics across the 3 regions (Table 2). Most LCs were female (97%) with a mean age ± SD of 48 ± 9.6 years. A majority of LCs were dieticians (94%) and health or diabetes educators (84.8%), with an average experience ± SD of 3.1 ± 2.02 years facilitating the program.

### Adoption

According to Proctor et al., adoption refers to experiences in the initial uptake and intention to try to implement the program. The group-DPP was first implemented in 2010 at 8 clinic sites: Region 1 (4 sites) and Region 2 (4 sites), and in 2011 at two additional sites (1 site in Region 2 and 1 site in Region 3). Through 2016, 10 more sites implemented GLB. However, between 2011 and 2019, five sites stopped offering the program (Additional file 2). Overall, 20 clinic sites across the regions implemented group-DPP (8 sites in Region 1, 10 sites in Region 2, and 2 sites in Region 3). Across all regions, the program has been, exclusively offered in English. The branded name of the program was chosen by region Directors, physician leads, and managers in each region. One region used the original “Group Lifestyle Balance™” trademarked name, whereas the other regions rebranded the program (names not disclosed to protect anonymity). In all regions, LCs indicated that the program was intended to serve as a weight management program and implementation was driven by a need to address increased prevalence of prediabetes and obesity in their populations (Table 3: Quotes 1.1 & 1.2).

**Table 3:** Qualitative Quotes

1. Adoption	1.1. “There is a higher need. We have a lot of patients in this area who need this kind of program” (7–1) 1.2. “We needed the program and we thought that it would be beneficial to help prevent diabetes and for weight loss” (34–3)
2. Penetration	2.1. “when I practice and I see a patient individually for weight management, I also will mention, we have this program that’s available to you” (20–2) 2.2. “I went to all of their staff meetings. I went to a staff meeting for every department in our area. So, I went and introduced myself and the program” (9–1) 2.3. “We had an endocrinologist here that was part of the program that pushed it and went around and talked to all the primary doctors about what a great program and the outcomes that Pittsburgh have had.” (23–2) 2.4. “The process is that we get a referral from the physician then they do a one-on-one consultation” (35–3) 2.5. “The providers are extremely busy. They do a follow up to a certain extent” (21–2) 2.6. “It falls off their radar when they’re managing everything else” (9–1)
3. Acceptability	3.1. “I love it when patients maybe lose a little bit of weight and change their eating lifestyle and then they come to me so excited that their lipid panels have improved or their A1C has improved” (34–3) 3.2. “It’s really allows patients to think, it gives them flexibility versus handing over a diet, so that’s good”(32–2) 3.3. “I think the strengths of the program were that it offered a proven program that worked” (28–2) 3.4. “I think it’s good information because they really need to have the medical evidence because the medical background made them understand that in the past this has worked for people and these are the statistics” (6–1) 3.5. “I do really like that that’s a big part of that program because it would get them thinking about why they made some of the choices that they made” (18–1) 3.6. “When we work on problem solving and just identifying emotional triggers, and then get into mindful eating; they’ve been like – it blows their mind. That stuff is powerful. I think that’s more important, honestly, than the nutritional info” (14–2) 3.7. “I think they found that when they have support from the other participants really helps keep them going to because they’re going to the same thing. I think it’s just the support they get in the program the kept them going” (35–3) 3.8. “I think having the group that was there cheered him” (2–2) 3.9. “No, it’s not because once we get the referral they can either choose to attend the one class Healthy Basics. If that doesn’t work out for them or they don’t want to do the class setting, then they get the one-on-one (35–3) 3.10. “It’s always a challenge to keep the group coherent and that one member doesn’t do all the talking. You have to control that group setting” (34–3) 3.11. “You don’t know who comes in the beginning, and if that group works, and if the chemistry of that group is working or not” (1–2) 3.12. “It’s presented in a manner that I think most people can understand and not feel overwhelmed by, especially when it’s explained” (22–2) 3.13. “The classes went in a nice order, kind of building on each topic throughout the three months” (12–1) 3.14. “Basically it puts people to sleep, so I make my own slides. I will get my own visual. I will bring in like food models, or real food and have them taste it” (33–2) 3.15. “They like the idea that they talk about a topic and they can set a goal based on that topic. Or, they’ll change a goal” (3–2) 3.16. “Aiming for 7% weight loss is often a lot less than what participants state that they want to lose …I think patients expect greater results” (27–2)
4. Appropriateness	4.1. “The patient population here is very educated, very smart. They’re technologically extremely advanced and savvy….they used to ask complex questions” (32–2) 4.2. “The highly educated that were in our groups, that was a negative because they felt it was just too simplistic and didn’t give them enough resources in their every-day life.” 25–2 4.3. “I think the learning level is appropriate. Because I think, the way that they break down the concepts is easy to understand for most people” (16–1) 4.4. “It’s written at a pretty good level…..the patients have all understood it pretty well” (34–3) 4.5. “I feel like it just skims the surface for a lot of information” (4–1) 4.6. “I think for my group… they can deal with more complicated examples.” (3–2) 4.7. “Some of the handouts just don’t have enough information about exercising or physical activity… whatever the topic may be” (15–2) 4.8. “It is very diet-oriented, which can backfire if you’re not careful” (24–2) 4.9. “It’s just kind of like … like nutrition, nutrition, nutrition, nutrition” (9–1) 4.10. “So, nutrition is constantly changing. Right? By the time you print something, the recommendation already changed…So, that was the problem with GLB, because that material we were looking at originally was like, 18 years ago maybe.” (15–2) 4.11. “it was just really targeting fat, so for some people, genetically they might do better with a different-- Maybe modifying carbs might work better for their body” (28–2)
5. Feasibility	5.1. “Right now, I don’t have a class so we are trying to waitlist people” (6–1) 5.2. “It’s not active at all in [302]. It was only offered once and that was it. It was poorly attended at that location, so we did not try it again for a second round. It has not been offered again.” (35–3) 5.3. “We don’t have facilitators. Yeah. I believe I’m the only one that was trained at this site to teach” (28–2) 5.4. “Now the [clinic site 1] closed up. We transferred patients to [clinic site 2]”
6. Fidelity	6.1. I’ll use, like, a PowerPoint for kind of like a virtual grocery shopping tour. When we talk about eating out, I’ll do a PowerPoint on menu comparisons” (8–1) 6.2. ” I make my own slides. I will get my own visual. I will bring in like food models, or real food and have them taste it. I incorporate a little game or something that people will get excited. So I do different things to get… if I see there’s an issue on certain things, I will look up on different apps because if it’s not covered, I will look it up myself.” 33–2″ 6.3. “I was also wanting to know what the participants wanted to learn. And so, I modified it according to their needs” (5–1) 6.4. “These people are pre-diabetic, so I have to tweak it; I cannot use that, having that it’s so many carbs. I’d be stoned. Yeah, so I have to tweak a lot of it” (33–2) 6.5. “I think a lot of people come in with a lot of knowledge. This area, people in general eat pretty healthily, or at least know how to. They already kind of had a fundamental knowledge; I think it was just adding onto it. They hear so much, read things on the internet, or they’ve already tried certain things, certain diets and so they come in with some baseline. So it’s just more of providing information on that level.” 13–2
7. Program Costs	7.1. “It depends on their insurance, we’ll kind of talk to them about their insurance coverage” (9–1). 7.2. “If they have an HMO and they attend nine out of 12 courses, they get reimbursed half….If they have Sutter Select and their BMI is over 30, then Sutter Select pays for it” (7–1) 7.3. “Most people find the cost pretty reasonable, but I have had some patients that, you know, they have to think about it for cost” (16–1) 7.4. “It’s a more rural area, more spread out. Most people or patients that I’ve spoken to, because insurance does not cover it for most part these types of programs, they can’t afford even that one day’s payment to come to a class” (18–1).
8. Sustainability	8.1. “I do talk to the physicians. Of course those that I see most frequently, they’re most familiar with the program. I have been to each and every physician’s office and have told them about this program” (17–1) 8.2. “Presenting the program at a standup meeting in the morning for all the providers to remind them that the class is starting in March and to remember who is an ideal candidate to refer” (22–2) 8.3. “We’ve switched to this rolling enrollment where somebody can start every month” (7–1) 8.4. “We do have a prompt pay discount, which we let them know about. If they pay in full within 30 days of receiving the bill, they can get, I think, a 30% reduction” (22–2) 8.5. “So we would touch base. If they were going to be there the next week I’d catch them up. Here’s written materials when they would come back again” (24–2) 8.6. “We did biweekly for the last three months instead of monthly. It seemed better. It’s easier to retain people, and they wanted it, too” (14–2) 8.7. “We’re screening to see if anybody has an underlying eating disorder. Then, also, psych history. We look at…We had some previous groups where there was patients with previous eating disorder behavior and a group—it wasn’t appropriate setting for them.” (25–2)

### Penetration

Penetration can be defined as the level of institutionalization and access to services. LCs were asked to describe program referral methods as well as visibility of the program to both patients and referring physicians. LCs from Regions 1 and 2 had similar perspectives on the visibility of the program to both patients and referring physicians. They made similar attempts at marketing to potential participants and to referring physicians (Table 3: Quotes 2.1 & 2.2). Region 2 relied on physicians who were outwardly supportive of the program, designated as “physician champions,” as a referral source for program participants (Table 3: Quote 2.3). However, LCs in Regions 1 and 2 stated that physicians’ referrals were inconsistent and generally insufficient. They described physicians’ awareness of the program as lacking due to physician turnover and competing care priorities (Table 3: Quotes 2.4–2.6). Physicians were the main source of referrals to the program in Region 3.

### Acceptability

Acceptability can be defined as LC’s satisfaction with various aspects of the program. LCs expressed their acceptance of the program overall, but there were notable differences in their perceptions across the three regions. All LCs believed that the program is effective in changing participants’ knowledge, attitudes, and behaviors towards healthy eating and physical activity and achieving positive health outcomes (Table 3: Quotes 3.1 & 3.2). They stated that the robust evidence base was a major strength of the program (Table 3: Quotes 3.3 & 3.4). In addition, LCs in Regions 1 and 2 highlighted as strengths the program’s focus on behavior change through the provision of problem-solving strategies and approaches to overcome emotional triggers (Table 3: Quotes 3.5 & 3.6).

Some LCs considered the group-based nature of the program a strength, providing accountability, motivation, peer support, and empowerment in helping participants to commit to the sessions and achieve positive outcomes (Table 3: Quotes 3.7 & 3.8). However, other LCs highlighted several challenges with the group-based structure of the program, including recruitment, enrollment, and implementation. They further stated that participants who are not comfortable with group classes would not enroll in the program (Table 3: Quote 3.9). In addition, they reported the lack of group cohesion, perceived to be driven by diversity in age and personalities among participants within the same group, as a barrier to program success (Table 3: Quotes 3.10 & 3.11).

LCs expressed mixed opinions regarding the curriculum design and content. In all regions they agreed that being simple, easy to follow, and well organized are strengths of the program (Table 3: Quote 3.12 & 3.13). However, there was general agreement that program materials were not visually appealing (Table 3: Quote 3.14).

Notable differences in LCs’ perspectives towards the program also were noted between regions in terms of goal setting and relevance of information included the curriculum. Some considered goal setting a strength of the program (Table 3: Quote 3.15), whereas others felt it could demotivate program participants and increase withdrawal, especially among those who either could not achieve their weekly goals or those who set ambitious weight loss goals (e.g. more than 10% of their initial body weight) (Table 3: Quote 3.16).

### Appropriateness

Appropriateness can be defined as the perceived fit, relevance and suitability of the program for the target population and participants. Differences in perceived fit were evident across regions. LCs at all sites in Region 2 perceived the curriculum’s content to be too basic for their patients, given higher underlying education levels of the population (Table 3: Quote 4.1 & 4.2). In Regions 1 and 3, with comparatively lower educational levels, the LCs considered the content to be appropriate, but information needed some tailoring to be relevant to their participants (Table 3: Quote 4.3- & 4.4). Further, several LCs within Regions 1 and 2 expressed that the curriculum lacked some important information (Table 3: Quote 4.5 &4.6), such as physical activity topics (Table 3: Quote 4.7), while overabundant in other topics such as nutrition information and calories. (Table 3: Quotes 4.8 & 4.9). The focus on calories and fat counting was perceived by virtually all LCs as inconsistent with newer nutritional science paradigms (Table 3: Quote 4.10 &4.11).

### Feasibility

Feasibility refers to the actual practicability or suitability for implementation of the program. The program was discontinued at a total of seven sites (4 sites in Region 2, 2 sites in Region 1, and 1 site in Region 3). Main reported reasons for discontinuing the program was increased patient attrition (2 sites in Region 1, 1 in Region 2 and 1 in Region 3). LCs across all three regions reported consistent difficulties with recruiting and retaining patients, which affected the sustainability of the program (Table 3: Quote 2.1 &5.2). s. Other reported reasons at two sites in Region 2 were related to lack of trained LCs to facilitate the program at the sites (Table 3: Quote 5.3) and change of location of the health education department where the program had been provided to another clinic site (1 site in Region 2) (Table 3: Quote 5.4).

### Fidelity

Fidelity can be defined as the extent to which the program or intervention is delivered as intended. Implementation of the program varied within and across regions in terms of LC training, eligibility criteria for participants, and program structure and delivery (Table 4). All LCs were trained before facilitating the program; however, types of training varied between regions. In Region 3, LCs received formal training from the University of Pittsburgh, whereas LCs in Region 1 were trained by their supervisor (i.e. peer-to-peer training), who received University of Pittsburgh certification for training new LCs. A variety of training modalities were used in Region 2: formal, in-person training from the University of Pittsburgh (61.9%), online or virtual training (19.1%), and peer-to-peer training (14.3%).

**Table 4:** Group Lifestyle Balance Component Description

GLB Standard Program Characteristics	Region 1	Region 2	Region 3
12 months program	12 months program	12 months program	3 months program
22 sessions in total	21 sessions	25 sessions in total	12 sessions in total
12 weekly intensive core sessions	12 weekly core sessions	13 weekly core sessions	12 weekly core sessions
4 biweekly transition sessions	No sessions	6 biweekly sessions	No sessions
6 monthly post-Core session	9 monthly Post-Core session	6 monthly Post-Core session	No Post-Core sessions
GLB Curriculum	GLB Curriculum	Modified GLB Curriculum	GLB Curriculum
Weekly Weigh-In	Yes	Yes	Yes
Continuous Self-tracking	Yes	Yes	Yes

Eligibility criteria varied within and across regions and differed from the initial target population of the DPP, that is, patients with clinical pre-diabetes or high risk of developing T2D. All regions targeted individuals who are overweight or obese, regardless of other risk factors and did not exclude individuals who already had a diabetes diagnosis. The majority of sites within Region 1 allowed all individuals to participate, regardless of diabetes risk. Eligibility criteria of patients referred to the program changed only in Region 2, whereby, over time, more diabetic patients had been included in the program after completing diabetes education classes. As a result of this difference in eligibility criteria, only approximately half of program participants meet DPP eligibility criteria (data not shown). Nearly 25% of participants have T2D, and 27% of participants are overweight or obese with no other risk factors for diabetes.

All regions offered group-based, in-person programs, consist with GLB™; however, variation in program structure were found across regions, including duration of the program, total number and frequency of sessions, and type of curriculum used. In Region 1, the duration of the program was 12 months. It included 12 core sessions provided on a weekly basis for approximately one hour, which is consistent with the GLB™ curriculum. In this region, however, post-core sessions were referred to as a “support group” and were not offered consistently at all sites due to attrition of patients enrolled in the program. The post-core sessions consisted of 9 optional sessions, provided once a month, for one hour each. In Region 3, the program consisted 12 core sessions delivered once per week for 3 months, corresponding to the intensive core phase of the GLB curriculum. In Region 2, the program initially consisted of 12 weekly core sessions; however, it changed in 2014 to a full-year program including 16 core sessions and 9 post core sessions. The 16 core sessions included 13 weekly sessions followed by 3 transitional biweekly sessions. The post-core sessions included 3 transitional sessions offered every other week followed by 6 monthly sessions.

All regions utilized the standardized GLB curriculum and content, with some pre-approved modifications included to allow for use among those with diabetes. These minor modifications (e.g. addressing hypoglycemia risk in the physical activity session) were made at the adoption stage and were approved by the University of Pittsburgh. Other micro-variations were observed across sites within Region 2 in terms of additions to the content (e.g. information about vitamins and supplements). LC’s used different types of visual aids to make the information more appealing (Table 3: Quotes 6.1 & 6.2). Since they first started providing the program at their sites, LC in both Regions 1 and 2 used the original GLB curriculum developed by the University of Pittsburgh. In 2014, three additional CDC-approved transitional sessions were incorporated to the curriculum used in Region 2, intended to help patients transition from weekly to monthly session schedule. The curriculum was also modified, with approval from the University of Pittsburgh, to include information for patients with a diabetes diagnosis (e.g. modified physical activity and diet recommendations). While LCs in Region 3 stated that they adhere to the curriculum materials, those in Regions 1 and 2 regularly provided additional and updated information to their patients. They elaborated on several topics included in the curriculum while modifying examples and providing additional strategies specific to diabetic patients in terms of calories, protein, and carbohydrates (Table 3: Quotes 6.3 & 6.4). However, LCs in Region 2 reported that they modified the level of information discussed and provided deeper explanations and examples to their patients who were perceived to be highly educated and knowledgeable about majority of the topics discussed in the curriculum (Table 3: Quote 6.5).

### Program costs

Implementation costs in Proctor’s framework refer to the marginal costs or perceived cost-effectiveness of the program as implemented. In this context, we refer to costs of the program to patient participants since we do not quantify the full cost of implementation from the institutional perspective. Cost of the 12-month program varied between regions. The costs of the program to patients was approximately 100% more expensive in Region 2 than Region 1, whereas the program was offered free of charge in Region 3, reflecting differences in socioeconomic status and ability to pay. However, in Region 1 the post-core sessions were considered optional and the patient was billed for each portion of the program separately. Insurance coverage of the program was not common and depended on an individual’s insurance plan and eligibility criteria (Table 3: Quotes 7.1 & 7.2). According to LCs in Region 1, the cost of the program was considered by some patients as fairly expensive and it was a major barrier to enrollment, especially those living in rural areas (Table 3: Quotes 7.3 & 7.4).

### Sustainability

Sustainability can be defined as the durability and institutionalization of the program to be maintained and implemented over time. To improve efficiency, LCs made several changes to the program in terms of recruitment and retention methods, as well as implementation of sessions and use of program materials. In Regions 1 and 2, LCs mentioned that informing and reminding physicians about the program increased patients’ referral to the program (Table 3: Quotes 8.1 & 8.2).

LCs used different strategies to increase patient recruitment, including rescheduling sessions to fill classes and referring patients to other nearby sites in case of lack of available spaces. To encourage patients to enroll in the program and prevent waiting lists, a rolling enrollment system was implemented in Region 1, whereby every week, participants could join the program at any core session (Table 3: Quote 8.3). In addition, prompt pay discounts or allowing for monthly or per-session payment were other strategies used in Region 2 to increase participants’ enrollment in the program (Table 3: Quote 8.4).

Participants’ attrition was reported as a challenge in all regions. In addition to following up and reaching out to their participants between classes, upfront payment was a retention strategy used in Region 1 and 2 (Table 3: Quote 8.5). However in Region 2, some LCs made changes to the frequency of post-core sessions from monthly to biweekly in an attempt to retain participants (Table 3: Quote 8.6). In addition, they applied strict pre-assessment requirements to recruit eligible and motivated patients who are ready to join the program (Table 3: Quote 8.7).

## Discussion

The integration of a group-DPP within a large healthcare delivery system, as a natural experiment, provides a unique opportunity to evaluate variation in implementation and to inform best practices for long-term success of this program in routine clinical settings across the nation. In this study, we observed instances of both consistency and variation in implementation of the program across three geographic regions, with very different underlying sociodemographic characteristics (Table 5). The findings of our study expose a dynamic and important tension between the benefits of fidelity to the original evidence-based program and tailoring the program to meet the local needs of the organization, distinct patient populations, and the clinical context.

**Table 5:** Regional Variation of Program Implementation by Implementation Domain

Domain	Consistent across all regions	Variable across regions
Adoption	• Promoted as a weight management program • Exclusively offered in English	• Branded name • Number of sites offering the program • Frequency of program offerings
Penetration	• Physician referrals as a recruitment method	• Visibility of program to patients • Consistency of physician referrals
Acceptability^a^	+ Evidence base of program + Easy-to-follow curriculum − Focus on calories and fat counting − Visual appeal of materials	• Program’s focus on behavior change • Group-based nature of program • Quality and level of physical activity and nutrition information in curriculum
Appropriateness	• No consistencies in views of appropriateness	• Suitability of curriculum’s educational level • Relevance of program material example stories and problems • Compatibility of program’s goal-setting guidelines
Feasibility	• Difficulties with recruitment and retention • Patient attrition as a reason for discontinuation at site	• Site also discontinued program due to other reasons (e.g. LC availability)
Fidelity	• Intensive core phase • Self-monitoring of food choices and weight • Content of post-core maintenance	• Type of training LC received • Eligibility criteria of participants • Variation in program structure (see Table 4 for details) • Supplementation of core curriculum with additional information for specific patient groups
Program Cost	• No consistencies in cost of program	• Cost of the program for participants • Insurance coverage • Perceived program affordability
Sustainability	• Patient attrition as a challenge	• Specific strategies to increase recruitment and stakeholder’s buy-in • Specific strategies to increase retention

^a^ + denotes acceptable; − denotes unacceptable

There is a dearth of studies that examine the real-world implementation of an evidence-based DPP. Moreover, this evaluation of implementation, under the conceptual framework described above, sets the stage for future work to examine the effect of implementation variation on outcomes. Several comments from LCs within different regions reflected differences in perceived appropriateness and acceptability and may have been influenced by underlying sociodemographic factors. The addition of content, visual aids, and other changes to the curriculum may have been an attempt on the part of LCs to increase appropriateness when it was perceived to be low. There is continued debate about the value of fidelity in implementation of existing evidence-based public health and clinical programs as originally developed versus adaptation of these program to make them more acceptable and applicable to a certain setting, population, or culture [29–32]. Fidelity can be defined as the degree to which an intervention is implemented as intended by its developers in order to ensure that the intervention remains effective [33]. It is described as the extent to which a program is implemented according to core elements included in the program manual such as: theoretical methods, strategies, determinants, target population, and activities delivered [29, 30]. Many of the core aspects and features of the group-based GLB program remained consistent and intact across all three regions over time. This included the use of the core curriculum and content for the intensive phase consisting of 12 weekly sessions. All clinics covered the core material of the program, with some LCs providing additional information and examples based on the perceived needs and demands of participants. Moreover, given that only clinics in Region 2 required the implementation of the post-core maintenance phase as compared to some clinics that did not offer it, or made it optional, the content of those sessions remained unchanged and consistent across regions. This was also the case with self-monitoring of food choices and weight, whereby LCs at all sites required and motivated program participants to track their calories intake and physical activities to achieve their lifestyle and weight loss goals. Other consistencies in implementation of the program were found across regions in terms of satisfaction with the evidence base (acceptability), referral methods (adoption), eligibility criteria (fidelity), and strategies to increase retention and effectiveness (sustainability).

Some aspects of the program were modified and changed, creating micro-adaptations to meet the needs of the distinct patient populations served by the region. For example, while difficulties with recruitment were common across regions (feasibility), strategies used to address these challenges differed (sustainability). Adaptation involves modifications made to the original design of an intervention during the implementation process and can include the adding, removing, or tailoring of information and/or activities from the original program [29, 34, 35]. While some studies have demonstrated that program adaptations can decrease effectiveness of the program [36–38], others argue that adaptation is necessary to increase stakeholder buy-in and improve the program’s delivery and relevance for the local target populations, while using available and accessible resources [39, 40]. Depending on the nature of modifications, adaptation could be beneficial or could threaten the theoretical basis of the intervention, resulting in a negative effect on expected outcomes. For example, some sites offered rolling admission to address recruitment challenges, allowing participants to begin that program midway through the session. While this may have improved recruitment numbers, the program and its content were originally designed to be sequential, building on foundational concepts as the program progresses. It remains unknown what impact this lack of fidelity, due to adaptation, may have on the ultimate effectiveness.

Studies exploring types of adaptations made to existing evidence-based programs showed that program providers modify programs based on various factors including target population needs [41], available physical and financial resources [42], and their own knowledge and expertise [43]. This was consistent with the results of our study showing that LCs supplemented the curriculum by providing updated information, based on their own knowledge, skills, and experiences, as well as their patients’ needs. In addition, facilitators “tweaked” and tailored examples within the curriculum to meet the population education level. The need for adaptation may be driven by the acceptability and/or perceived appropriateness of the intervention. The underlying patient populations in each region are very different in terms of sociodemographic and race/ethnicity. Additions, omissions, and other changes to the curriculum were likely influences by the LC perception appropriateness for a given patient population. It is unknown how adaptations and variation in implementation ultimately influence effectiveness or clinical outcomes. While we did not examine this in the present study, future studies are underway to examine how each of these implementation factors may influence patient outcomes. This information will be crucial to resolving the tension between optimizing the benefits of both fidelity and adaptation as needed and appropriate for a given patient population.

While many of the issues described are not unique to the health system in question, this was a qualitative study of 33 LCs from a single healthcare system in the western U.S. However, this healthcare delivery system, as a mixed-payer, FFS provider organization, is similar to most other healthcare settings in the nation. Thus, these findings have the potential for broad applicability. Given that the views and perspectives are based on individual responses, other health systems should consider examining program implementation of group-DPP to validate these findings. Further, while it is important to examine the ways in which program implementation was consistent or varies across sites, future research is needed to explore how this these factors may serve as barriers or facilitators to successful implementation of the program. Finally, the program was first implemented nearly a decade ago, which may introduce recall bias. Natural experiments provide an opportunity to study interventions in real-world clinical settings, yet such experiments have several challenges, including non-standardized metrics, inconsistent data collection over time, and incomplete data capture, which has limited our ability to determine the true impact of implementation factors, such as loss of fidelity or low perceived appropriateness, on goal attainment among participants.

Healthcare leadership and program implementers must consider building evaluation into the intervention prior to implementation, in order to be able to assess effectiveness and impact of the program. The CDC’s National DPRP provides some incentive for standardized data collection and evaluation, as recognition status is linked to the achievement of several requirements and performance metrics for a given program. More work is needed to support efficient yet robust data collection for program evaluation within healthcare systems.

## Conclusions

Here we provide an in-depth examination of differences in program implementation and local adaptations in structure and design of group-DPP in a real-world healthcare setting. There were instances of both consistency and variation in implementation of the program across three geographic regions, revealing a dynamic and important tension between the desire to retain fidelity to the original evidence-based program and the need to tailor the program to meet the local needs of the organization, distinct patient populations, and the clinical context. In addition, certain challenges present consistently across sites, in particular, challenges resulting from a reliance on physician referrals as a method of recruitment, and challenges of patient attrition over the course of the year-long program. Identifying the common challenges faced across sites also offers opportunities for considering alternative approaches to implementation to anticipate these potential barriers. Findings may inform national diabetes prevention guidelines, such as the DPRP initiative, for facilitating successful adoption and long-term sustainability of programs. Further research is needed to explore how differences in implementation domains impact program effectiveness.

## Supplementary information

**Additional file 1.** Participant Socio-demographics and Baseline Characteristics. Bivariate analysis of program participants sociodemographic and baseline characteristics across the three geographic regions.

**Additional file 2.** Active Group-Based Diabetes Prevention Program Sites. Number of clinic sites providing the program, per year (from 2010 until 2019), at each of the three geographic regions.

## Data Availability

The dataset, which includes participants’ transcripts, is not publicly available due to confidentiality policies.

## Abbreviations

CDC: Centers for Disease Control and Prevention
CVD: Cardiovascular diseases
DPP: Diabetes Prevention Programs
DPRP: Diabetes Prevention Recognition Program
GLB: Group Lifestyle Balance™
Group-DPP: Group-based versions of the DPP
LC: Lifestyle coaches
NIDDK: National Institute of Diabetes and Digestive and Kidney Diseases
SD: Standard deviation
T2D: Type 2 diabetes
U.S: United States

## References

[1] National Diabetes Statistics Report, 2017. Centers for Disease Control and Prevention. Atlanta: Centers for Disease Control and Prevention (CDC); 2017.

[2] Pan XR (1997). Effects of diet and exercise in preventing NIDDM in people with impaired glucose tolerance. The Da Qing IGT and diabetes study. Diabetes Care.

[3] Economic Costs of Diabetes in the U.S. in 2017. Diabetes Care, 2018: p. dci180007. 10.2337/dci18-0007 PMC5911784 29567642

[4] Trikkalinou A, Papazafiropoulou AK, Melidonis A (2017). Type 2 diabetes and quality of life. World J Diabetes.

[5] Weiss EP (2006). Improvements in glucose tolerance and insulin action induced by increasing energy expenditure or decreasing energy intake: a randomized controlled trial. Am J Clin Nutr.

[6] Brink S (2009). The diabetes prevention program: how the participants did it. Health Aff (Millwood).

[7] Knowler WC (2002). Reduction in the incidence of type 2 diabetes with lifestyle intervention or metformin. N Engl J Med.

[8] Greenwood DA (2014). Adapting the group lifestyle balance program for weight management within a large health care system diabetes education program. Diabetes Educ.

[9] Diabetes Prevention Program Research, G (2002). The Diabetes Prevention Program (DPP): description of lifestyle intervention. Diabetes Care.

[10] Betts AC, Froehlich-Grobe K (2017). Accessible weight loss: adapting a lifestyle intervention for adults with impaired mobility. Disabil Health J.

[11] Kriska A (2003). Can a physically active lifestyle prevent type 2 diabetes?. Exerc Sport Sci Rev.

[12] Kriska AM (2006). Physical activity in individuals at risk for diabetes: diabetes prevention program. Med Sci Sports Exerc.

[13] Jiang L (2013). Translating the diabetes prevention program into American Indian and Alaska native communities: results from the special diabetes program for Indians diabetes prevention demonstration project. Diabetes Care.

[14] Davis-Smith YM (2007). Implementing a diabetes prevention program in a rural African-American church. J Natl Med Assoc.

[15] Driver S, Reynolds M, Kramer K (2017). Modifying an evidence-based lifestyle programme for individuals with traumatic brain injury. Brain Inj.

[16] Ackermann RT (2008). Translating the diabetes prevention program into the community. The DEPLOY pilot study. Am J Prev Med.

[17] Kramer MK (2009). Translating the diabetes prevention program: a comprehensive model for prevention training and program delivery. Am J Prev Med.

[18] Aziz Z (2015). A systematic review of real-world diabetes prevention programs: learnings from the last 15 years. Implement Sci.

[19] Venditti EM, Kramer MK (2013). Diabetes prevention program community outreach: perspectives on lifestyle training and translation. Am J Prev Med.

[20] Baker MK (2011). Behavioral strategies in diabetes prevention programs: a systematic review of randomized controlled trials. Diabetes Res Clin Pract.

[21] Kramer MK (2018). Evaluation of a diabetes prevention program lifestyle intervention in older adults: a randomized controlled study in three senior/community centers of varying socioeconomic status. Diabetes Educ.

[22] Kanaya AM (2012). The live well, be well study: a community-based, translational lifestyle program to lower diabetes risk factors in ethnic minority and lower-socioeconomic status adults. Am J Public Health.

[23] Albright AL, Gregg EW (2013). Preventing type 2 diabetes in communities across the U.S.: the National Diabetes Prevention Program. Am J Prev Med.

[24] Kramer MK (2011). A community-based diabetes prevention program: evaluation of the group lifestyle balance program delivered by diabetes educators. Diabetes Educ.

[25] Proctor E (2011). Outcomes for implementation research: conceptual distinctions, measurement challenges, and research agenda. Admin Pol Ment Health.

[26] Dedoose 2018; Version 8.0.35 [Web application for managing, analyzing, and presenting qualitative and mixed method research data]. Available from: www.dedoose.com.

[27] Race/Ethnicity of Individual, 2016 American Community Survey 1-year estimates. 2016, US Census Bureau.

[28] Income/Earnings (Households), 2016 American Community Survey 1-year estimates. 2016, US Census Bureau.

[29] Owczarzak J, Broaddus M, Pinkerton S (2016). A qualitative analysis of the concepts of fidelity and adaptation in the implementation of an evidence-based HIV prevention intervention. Health Educ Res.

[30] Berman P, McLaughlin MP (1976). Implementation of educational outcomes. Educ Forum.

[31] Boruch RR, Gomez H (1977). Sensitivity, bias, and theory in impact evaluation. Prof Psychol.

[32] Bopp M, Saunders RP, Lattimore D (2013). The tug-of-war: fidelity versus adaptation throughout the health promotion program life cycle. J Prim Prev.

[33] Pérez D, Van der Stuyft P, Zabala MC, Castro M, Lefèvre P. A modified theoretical framework to assess implementation fidelity of adaptive public health interventions. Implement Sci. 2016;11(1):91. 10.1186/s13012-016-0457-8 PMC4939032 27391959

[34] Cunningham SD, Card JJ (2014). Realities of replication: implementation of evidence-based interventions for HIV prevention in real-world settings. Implement Sci.

[35] Moore JE, Bumbarger BK, Cooper BR (2013). Examining adaptations of evidence-based programs in natural contexts. J Prim Prev.

[36] Durlak JA, DuPre EP (2008). Implementation matters: a review of research on the influence of implementation on program outcomes and the factors affecting implementation. Am J Community Psychol.

[37] Dane AV, Schneider BH (1998). Program integrity in primary and early secondary prevention: are implementation effects out of control?. Clin Psychol Rev.

[38] McKleroy VS (2006). Adapting evidence-based behavioral interventions for new settings and target populations. AIDS Educ Prev.

[39] Harshbarger C (2006). An empirical assessment of implementation, adaptation, and tailoring: the evaluation of CDC's National Diffusion of VOICES/VOCES. AIDS Educ Prev.

[40] Rohrbach LA (2006). Type II translation: transporting prevention interventions from research to real-world settings. Eval Health Prof.

[41] Galbraith JS, Stanton B, Boekeloo BEA (2008). Exploring implementation and fidelity of evidence-based behavioral interventions for HIV preventions: lessons learned from the focus on kids diffusion case study. Health Educ Behav.

[42] Payne AA, Eckert R (2010). The relative importance of provider, program, school, and community predictors of the implementation quality of school-based prevention programs. Prev Sci.

[43] Palinkas LA (2008). An ethnographic study of implementation of evidence-based treatments in child mental health: first steps. Psychiatr Serv.

