# Supplementary material for: Implementation of a group-based diabetes prevention program within a healthcare delivery system
Source: BMC Health Serv Res. 2019 Oct 15;19:694. doi: 10.1186/s12913-019-4569-0 (PMC6792249; doi:10.1186/s12913-019-4569-0)
Supplement: Supplementary file 1 — Additional file 1. Participant Socio-demographics and Baseline Characteristics. Bivariate analysis of program participants sociodemographic and baseline characteristics across the three geographic regions. [file 12913_2019_4569_MOESM1_ESM.docx]

**Additional File 1: Participant Socio-demographics and Baseline Characteristics**

|  | **Region 1**  **N = 2040** | **Region 2  N = 961** | **Region 3**  **N = 155** | **P-Value** |
| --- | --- | --- | --- | --- |
| **PARTICIPANT DEMOGRAPHICS** |  |  |  |  |
| **Mean age, years ± SD** | 53.53±13.50 | 53.07±12.30 | 55.15±12.52 | 0.1757 |
| **Female, n (%)** | 1572 (77.06) | 759 (78.98) | 121 (78.06) | 0.4955 |
| **Race/ethnicity, n (%)** |  |  |  | <0.0001* |
| African American | 110 (5.39) | 23 (2.39) | 7 (4.52) |  |
| Asian | 90 (4.41) | 88 (9.16) | 2 (1.29) |  |
| Hispanic | 234 (11.47) | 118 (12.28) | 39 (25.16) |  |
| Non-Hispanic White | 1447 (70.93) | 607 (63.16) | 95 (61.29) |  |
| Other | 57 (2.79) | 7 (0.73) | 8 (5.16) |  |
| Unknown | 102 (5.00) | 118 (12.28) | 4 (2.58) |  |
| **English proficient, n (%)** | 2003 (98.19) | 927 (96.46) | 150 (96.77) | 0.0127* |
| **Insurance payer, n (%)** |  |  |  | <0.0001* |
| Commercial FFS/PPO | 785 (38.48) | 569 (59.21) | 68 (43.87) |  |
| Commercial HMO | 321 (15.74) | 218 (22.68) | 21 (13.55) |  |
| Medicare (FFS/ HMO) | 410 (20.10) | 158 (16.44) | 45 (29.03) |  |
| Medicaid | 48 (2.35) | 7 (0.73) | 2 (1.29) |  |
| Other/ Self/ Unknown | 476 (23.33) | 9 (0.94) | 19 (12.26) |  |
| **Median household income, n (%)** |  |  |  | <0.0001* |
| <$50,000 | 258 (12.65) | 35 (3.64) | 60 (38.71) |  |
| ≥$50,000 to <$75,000 | 1074 (52.65) | 285 (29.66) | 92 (59.35) |  |
| ≥$75,000 to <$100,000 | 660 (32.35) | 351 (36.52) | 2 (1.29) |  |
| ≥$100,000 | 48 (2.35) | 290 (30.18) | 1 (0.65) |  |
| **CLINICAL CHARACTERISTICS** |  |  |  |  |
| **Mean weight, kg ± SD** | 102.61±24.66 | 98.35±21.63 | 102.93±25.01 | <0.0001* |
| Weight missing, n (%) |  |  |  |  |
| **BMI categories, n (%)** |  |  |  | 0.0375* |
| Normal | 32 (1.57) | 15 (1.56) | 2 (1.29) |  |
| Overweight | 276 (13.53) | 158 (16.44) | 22 (14.19) |  |
| Obese | 1156 (56.67) | 570 (59.31) | 84 (54.19) |  |
| Severe Obese | 576 (28.24) | 218 (22.68) | 47 (30.32) |  |
| **COMORBIDITIES** |  |  |  |  |
| **Prediabetes, n (%)** | 192 (9.41) | 200 (20.81) | 30 (19.35) | <0.0001* |
| **T2D, n (%)** | 546 (26.76) | 165 (17.17) | 44 (28.39) | <0.0001* |
| **Hypertension, n (%)** | 946 (46.37) | 373 (38.81) | 72 (46.45) | 0.0004* |
| **Dyslipidemia, n (%)** | 834 (40.88) | 452 (47.03) | 94 (60.65) | <0.0001* |
| **Metabolic syndrome, n (%)** | 504 (24.71) | 220 (22.89) | 48 (30.97) | 0.0864 |
| **ASCVD, n (%)** | 143 (7.01) | 49 (5.10) | 22 (14.19) | 0.0001* |
| **No prediabetes, diabetes or metabolic syndrome, n (%)** | 1266 (62.06) | 582 (60.56) | 79 (50.97) | 0.0224* |
| **Depression, n (%)** | 428 (20.98) | 208 (21.64) | 25 (16.13) | 0.2928 |
| **CCI Score** |  |  |  | <0.0001* |
| 0 | 1063 (52.11) | 599 (62.33) | 67 (43.23) |  |
| 1-2 | 835 (40.93) | 333 (34.65) | 72 (46.45) |  |
| 3-4 | 117 (5.74) | 26 (2.71) | 14 (9.03) |  |
| 5-6 | 22 (1.08) | 2 (0.21) | 1 (0.65) |  |
| >6 | 3 (0.15) | 1 (0.10) | 1 (0.65) |  |
