# Supplementary material for: Implementation of a group-based diabetes prevention program within a healthcare delivery system
Source: BMC Health Serv Res. 2019 Oct 15;19:694. doi: 10.1186/s12913-019-4569-0 (PMC6792249; doi:10.1186/s12913-019-4569-0)
Supplement: Supplementary file 2 — Additional file 2. Active Group-Based Diabetes Prevention Program Sites. Number of clinic sites providing the program, per year (from 2010 until 2019), at each of the three geographic regions. [file 12913_2019_4569_MOESM2_ESM.docx]

**Additional File 2: Active Group-Based Diabetes Prevention Program Sites**

| **Number of Active Site** | **2010-2011** | **2011-2012** | **2012-2013** | **2013-2014** | **2014-2015** | **2015-2016** | **2016-2017** | **2017-2018** | **2018-2019** |
| --- | --- | --- | --- | --- | --- | --- | --- | --- | --- |
| **Region 1** | 4 | 4 | 5 | 6 | 7 | 8 | 8 | 8 | 8 |
| **Region 2** | 4 | 5 | 4 | 4 | 8 | 8 | 9 | 8 | 6 |
| **Region 3** | 0 | 1 | 1 | 2 | 1 | 1 | 1 | 1 | 1 |
| **Total** | 8 | 10 | 10 | 12 | 16 | 17 | 18 | 17 | 15 |
